# Antiviral Mechanisms of Justicidin B and Tuberculatin from *Phyllanthus brasiliensis* against Oropouche Virus Infection

**DOI:** 10.1021/acsomega.6c00627

**Published:** 2026-05-19

**Authors:** Marília Bueno da Silva Menegatto, Ariane Coelho Ferraz, Rafaela Lameira Souza Lima, Allen René Ruiz Hernández, Pedro Henrique Guimarães, Pedro Alves Machado-Junior, Paulo Wender P. Gomes, Sônia das Graças Santa Rosa Pamplona, Edina Raquel Meneses Silva, Ellen Gonçalves de Oliveira, Adriana Cotta Cardoso Reis, José Carlos de Magalhães, Geraldo Célio Brandão, Jordana Grazziela Alves Coelho-dos-Reis, Erna Geessien Kroon, Consuelo Yumiko Yoshioka e Silva, Milton Nascimento da Silva, Cintia Lopes de Brito Magalhães

**Affiliations:** † Biological Sciences Post-Graduation Program, Center for Research in Biological Sciences, Federal University of Ouro Preto, Ouro Preto, Minas Gerais 35400-000, Brazil; ‡ Chemistry Post-Graduation Program, Institute of Exact and Natural Sciences, Federal University of Pará, Belém, Pará 66075-110, Brazil; § Biodiversity and Biotechnology Post-Graduation Program (BIONORTE-Pará), Federal University of Pará, Belém, Pará 66075-110, Brazil; ∥ Pharmaceutical Sciences Post-Graduation Program, Institute of Health Sciences, Federal University of Pará, Belém, Pará 66075-110, Brazil; ⊥ Microbiology Post-Graduation Program, Institute of Biological Sciences, Federal University of Minas Gerais, Belo Horizonte, Minas Gerais 31270-901, Brazil; # Pharmaceutical Sciences Post-Graduation Program, School of Pharmacy, Federal University of Ouro Preto, Ouro Preto, Minas Gerais 35400-000, Brazil; ∇ Biotechnology Post-Graduation Program, Federal University of São João del-Rei, São João del-Rei, Minas Gerais 36307-352, Brazil; ○ Biotechnology Post-Graduation Program, Center for Research in Biological Sciences, Federal University of Ouro Preto, Ouro Preto, Minas Gerais 35400-000, Brazil

## Abstract

Oropouche virus (OROV) has emerged as a growing public health concern in Latin America, with an unprecedented surge in Oropouche fever cases since 2023. Previously considered a sporadic and neglected arboviral infection, Oropouche fever now represents one of the most rapidly expanding arboviral threats in the region. Despite its increasing incidence, no approved vaccines or antiviral therapies are available, highlighting the urgent need for effective interventions. Natural compounds, particularly those derived from *Phyllanthus brasiliensis*, have shown promising antiviral properties against several RNA viruses. This study investigated the anti-OROV activity and mechanism of action of two lignans isolated from *P. brasiliensis*, justicidin B and tuberculatin. Cytotoxicity was evaluated by MTT assay, and antiviral efficacy was quantified by plaque-forming assays. Mechanistic analyses included virucidal assays and evaluations at distinct stages of the viral replication cycle. Both lignans exhibited potent anti-OROV activity, with selectivity indices of 2307 (justicidin B) and 1153 (tuberculatin). Chloroquine was used as a positive control and was found to be 47.7 and 30.1 times less potent than justicidin B and tuberculatin, respectively. Treatments initiated within 6 hpi reduced viral titers by >99.99% (>4log_10_), and by ∼2log_10_ when added at 12 hpi. These results demonstrate that justicidin B and tuberculatin act mainly in intracellular processes occurring after viral entry. Molecular coupling analysis corroborated these findings, as both demonstrated a propensity to interact with residues of the OROV Gc glycoprotein, highlighting their strong therapeutic potential as lead candidates for antiviral development against Oropouche fever.

## Graphical abstract

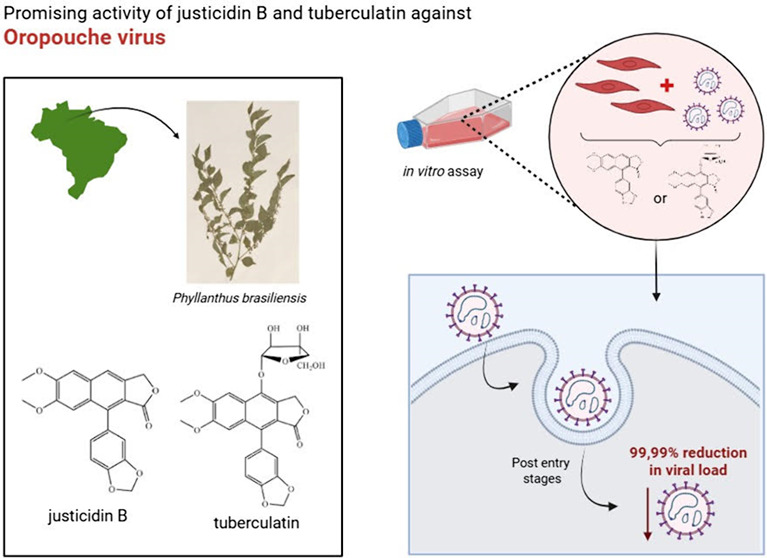

## 1 Introduction

The *Orthobunyavirus oropoucheense*, a member of the family *Peribunyaviridae* and the genus *Orthobunyavirus*, is the arbovirus responsible for causing Oropouche fever (OF). Oropouche virus (OROV) is an enveloped virus with a tripartite genome composed of three single-stranded, negative-sense RNA segments (S, small; M, medium; and L, large). It is primarily transmitted to humans through the biting midges (*Culicoides paraensis*). It was first isolated in 1955 in Trinidad and Tobago and has since infected over half a million people.^1^

Until 2022, OROV had been detected in South America (primarily northern Brazil) and parts of Central America and the Caribbean, causing periodic local outbreaks of febrile illness.^2^ Nevertheless, by the end of 2023, OROV had reached proportions never seen before in the history of OF. OROV was responsible for causing large outbreaks in endemic areas, however this outbreak also marks the first time that nonendemic regions in Latin America have registered cases of local infection, including Brazil (states such as Bahia, Ceará, Pernambuco, Piauí, Espírito Santo, Minas Gerais, Rio de Janeiro, Mato Grosso, Mato Grosso do Sul and Santa Catarina),^3^ Peru, Colombia, Cuba and Bolivia.^4^ Furthermore, in Brazil, more than 13 thousand cases of OROV were confirmed in 2024, and in 2025 (from January to June) more than 11 thousand.^5,6^ In 2025, to date, 4 deaths associated with OROV infection have been identified, in Espírito Santo (1) and Rio de Janeiro (3).^6^ In addition, it has been confirmed that OROV can infect the placenta and fetus, causing congenital infection and adverse perinatal outcomes, such spontaneous abortion, stillbirth, and congenital anomalies including central nervous system (CNS) involvement with microcephaly.^1^

The symptoms of OF are indistinguishable from other febrile arboviruses, such as fever, headache, retro-orbital pain, chills, malaise, myalgia and arthralgia. Severe symptoms may include vomiting and bleeding, such as petechiae, epistaxis, and gingival bleeding. Cases involving CNS may progress to meningitis or encephalitis.^3^ Despite all its epidemiological importance and the recent increase in the number of cases reported in outbreaks, there are still no vaccines or antivirals available for OROV,^7^ in which related studies are also scarce.

In this sense, natural products have been widely used as a resource for the development of new antiviral drugs.^8^ More specifically, plants of the genus *Phyllanthus* (Phyllanthaceae) have been frequently used in folk medicine to treat different diseases. In the Brazilian Amazon, *Phyllanthus brasiliensis* (Aubl.) Poir. also known as “quebra-pedra” has interesting pharmacological properties. Among them, our research group previously reported the antiviral activity of *P. brasiliensis* extracts against medical importance arboviruses: Mayaro, Chikungunya, Zika, including OROV.^9^ This significant antiviral activity demonstrated by these extracts instigates the investigation of the mechanisms of action and interaction of the compounds present with the viral multiplication cycle, such as viral entry, replication, assembly and release. In this report, we aimed to elucidate the antiviral mechanisms against OROV of two compounds isolated from *P. brasiliensis*: justicidin B and tuberculatin.

## 2 Results

### 2.1 Isolated Compounds

Two lignans isolated from the leaves of *P. brasiliensis* were selected for evaluation of anti-OROV activity. The structures of justicidin B (1) (97% purity) and tuberculatin (2) (95% purity) are shown in Figure 1. More details on compound characterization can be found at Carvalho et al.^9^

**Figure 1:**
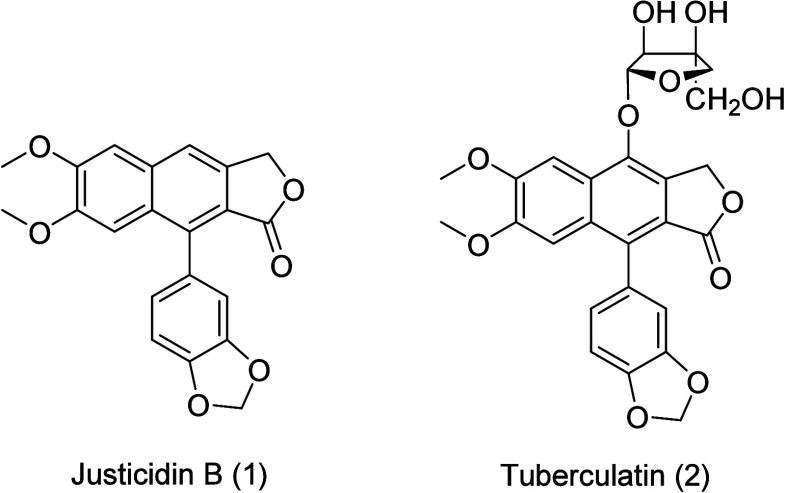
Structures of the lignan justicidin B and glycosylated lignan tuberculatin isolated from leaves of *Phyllanthus brasiliensis*.

### 2.2 Justicidin B and Tuberculatin Protect Cells from Infection

The cytotoxicity assay showed that neither the highest concentration of justicidin B nor tuberculatin was able to reduce cell viability by 50% (Figure 2). Furthermore, no toxicity was observed when Vero cells were treated with DMSO vehicle alone (data not shown).

**Figure 2:**
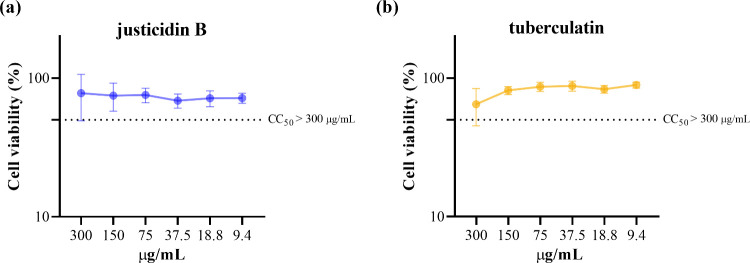
Cytotoxicity of justicidin B and tuberculatin in Vero cells. Cell viability was assessed after 48 h of treatment with different concentrations of justicidin B (a) and tuberculatin (b). The dotted line indicates the cytotoxic concentration for 50% of the cells (CC_50_). Data are presented as mean ± standard error of the mean (SEM) for all experiments.

When evaluating antiviral activity, both justicidin B and tuberculatin were shown to effectively protect cells from OROV infection. The protection induced by lignan and glycosylated lignan was shown to be dose-dependent, in which low concentrations of both were necessary to cause 50% cellular protection. The calculated EC_50_ values were equal to 0.36 ± 0.10 μg/mL and 0.57 ± 0.18 μg/mL for justicidin B (Figure 3a) and tuberculatin (Figure 3b), respectively.

**Figure 3:**
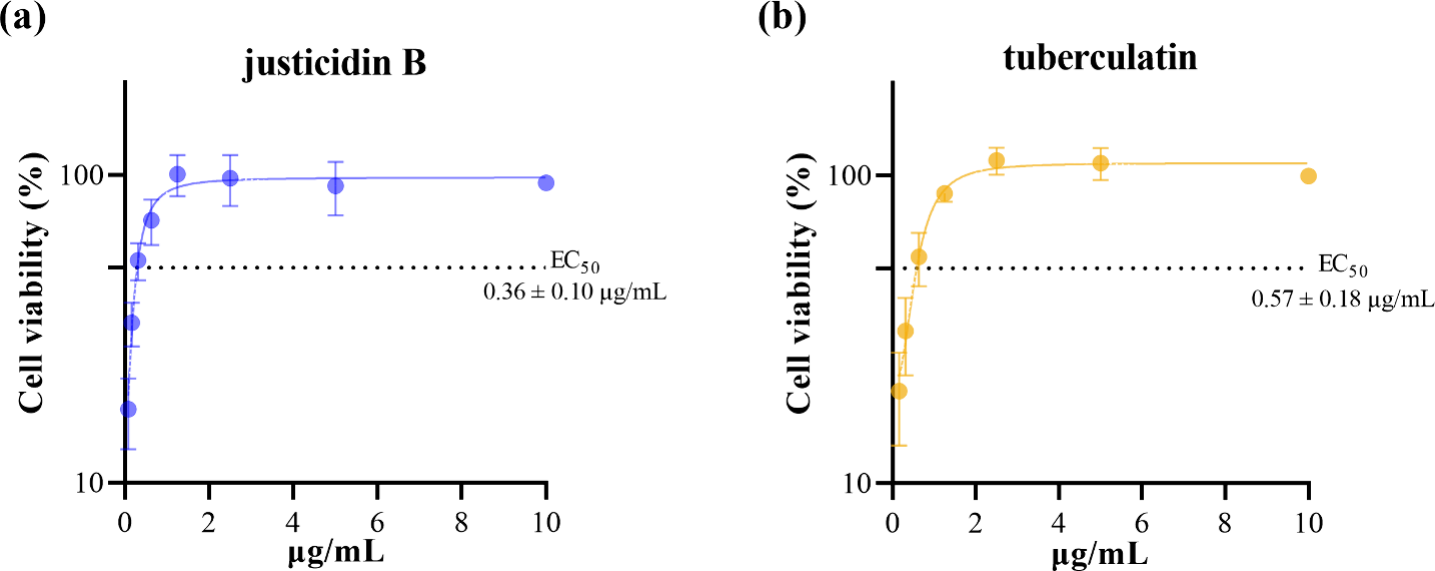
Anti-OROV activity of justicidin B and tuberculatin. Cell viability after 48 h of OROV infection (moi 1) and treatment with different concentrations of justicidin B (a) and tuberculatin (b). The dotted line indicates the effective concentration for 50% of the cells (EC_50_). Data are presented as mean ± standard error of the mean (SEM) for all experiments.

Chloroquine was used as the positive control, exhibiting a CC_50_ value of 41.94 ± 1.29 μg/mL and an EC_50_ value of 17.16 ± 2.52 μg/mL. These results indicate that justicidin B and tuberculatin were 47.7- and 30.1-fold more potent than chloroquine, respectively, in protecting cells from OROV infection. Moreover, the antiviral evaluation of the DMSO vehicle (negative control) did not show any inhibitory action against OROV (data not shown), confirming the absence of influence on the bioactivity of the compounds under study.

After the incubation period of the antiviral assay, it can be noted that the cells infected with OROV and treated with tuberculatin (Figure 4c) or justicidin B (Figure 4d) at concentrations higher than EC_50_ presented a morphology very similar to the uninfected and untreated cells (control, Figure 4a), in which few dead cells were observed, maintaining the natural arrangement of fibroblasts. The potent antiviral effect of the compounds isolated from *P. brasiliensis* against OROV becomes even more evident when the infected and treated cells are compared to the cells that were only infected and untreated (viral control, Figure 4b). In viral control, it is possible to observe the absence of living cells, whose viral cytopathic effect is very pronounced, and the cells no longer have the arrangement of fibroblasts. Thus, it can be stated that justicidin B and tuberculatin are capable of protecting cells from death induced by OROV infection. In addition, optical microscopy confirms the results presented in Figure 4, in which cell viability was measured indirectly by the colorimetric assay with MTT.

**Figure 4:**
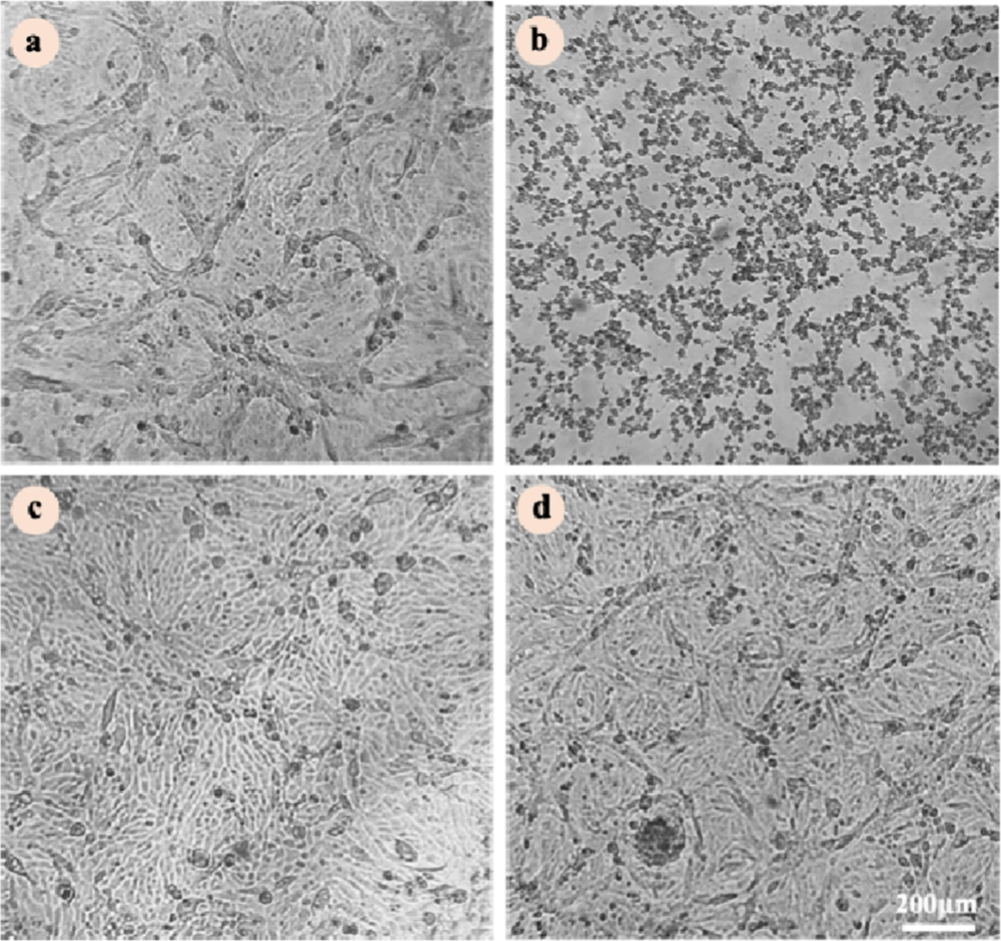
Justicidin B and tuberculatin protect cells from OROV infection. Cell monolayer microscopy after 48 h of incubation with virus and compounds. (a) control cells—untreated and uninfected cells. (b) viral control—infected and untreated cells. (c) infected and treated cells with 5 μg/mL tuberculatin. (d) cells infected and treated with 5 μg/mL justicidin B. Images captured at a magnification of 100×.

### 2.3 Justicidin B and Tuberculatin Reduce Viral Load after Treatment

Viral load of supernatant from antiviral assay cells was assessed after treatment with justicidin B or tuberculatin. Results showed the same inhibitory behavior for both: at a concentration of 10 μg/mL (above the EC_50_), an inhibition of 99.999% was observed, reducing almost 6 log_10_ when compared to the viral control (Figure 5). The reduction in viral load after treatment with *P. brasiliensis* isolates was also dose-dependent. Moreover, concentrations close to the EC_50_ of both justicidin B (0.31 μg/mL) and tuberculatin (0.62 μg/mL) resulted in an inhibition of almost 90% of the viral load. The IC_50_ of each compound was determined from the viral load inhibition data for each concentration evaluated. Justicidin B presented an IC_50_ of 0.13 ± 0.03 μg/mL and tuberculatin of 0.26 ± 0.15 μg/mL. It is important to emphasize that it was not evaluated whether treatment with concentrations above 10 μg/mL of justicidin B or tuberculatin would result in a total reduction in viral load.

**Figure 5:**
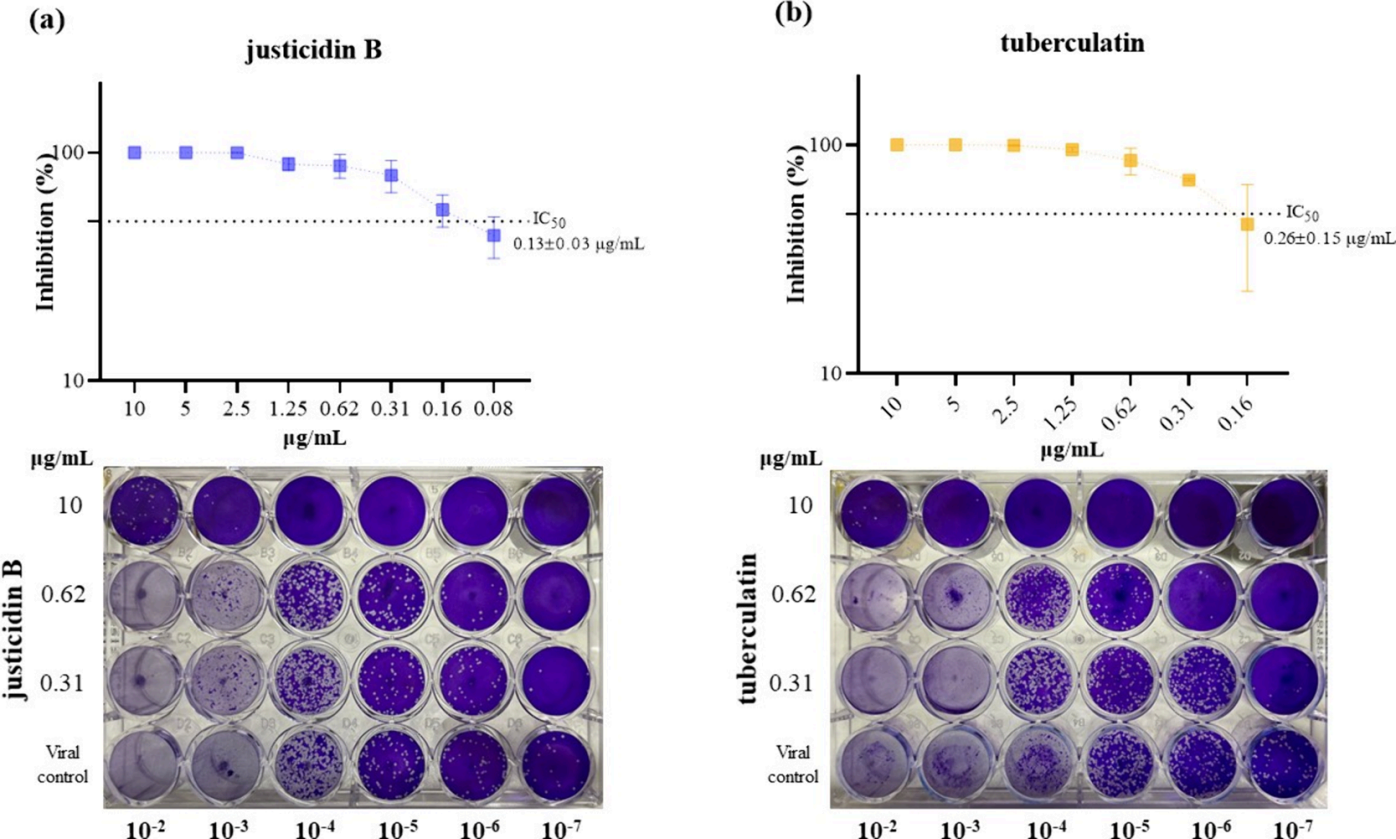
Justicidin B and tuberculatin reduce viral load after treatment. Viral quantification of supernatant from cells infected by OROV and treated with (a) justicidin B and (b) tuberculatin, and photo of the plaques showing the potent inhibitory effect against OROV of compounds. Data are presented mean ± mean standard error (SEM).

The SI of the two lignans (also known as arylnaphthalenes) under study was calculated from the CC_50_ and IC_50_ values (Table 1). Justicidin B and tuberculatin presented an SI of 2307 and 1153, respectively. These high values found demonstrate the high selectivity of the compounds, which can significantly inhibit OROV without causing toxicity to the host cell.

**Table 1:** Cytotoxicity and Anti-Oropouche Virus Activity of Isolated Compounds of *Phyllanthus brasiliensis* ^a^

substance	CC_50_ (μg/mL)^b^	EC_50_ (μg/mL)^c^	IC_50_ (μg/mL)^d^	SI^e^
justicidin B	>300	0.36 ± 0.10	0.13 ± 0.03	2307.69
tuberculatin	>300	0.57 ± 0.18	0.26 ± 0.15	1153.85
chloroquine	41.94 ± 1.29	17.16 ± 2.52	–^f^	–^f^

a All data were obtained from three independent experiments with three replicates each.

b Cytotoxic concentration for 50% of cells.

c Effective concentration that protects 50% of cells.

d Inhibitory concentration that reduces 50% of viral load.

e Selectivity Index: ratio between substance’s CC_50_ and IC_50_.

f –: Not determined.

### 2.4 Justicidin B and Tuberculatin Inhibit OROV in Postinfection Intracellular Stages

Given the high cellular protection, selectivity, and reduction of viral load found for justicidin B and tuberculatin, we began the search for the mechanism of antiviral action of these compounds. When evaluating the virucidal activity, we observed that neither justicidin B nor tuberculatin can reduce the viral load significantly (Figure 6a), showing that the antiviral action of both compounds is not due to direct interaction with the viral particle. The compounds also did not show a significant inhibitory effect when the treatment was performed only in the viral adsorption stage (Figure 6b), evidencing that justicidin B and tuberculatin are not able to inhibit OROV entry receptors into the host cell. In the internalization stage, only the treatment with justicidin B was able to reduce the viral load significantly (Figure 6c), with inhibition of 99% of the viral load (∼2 log_10_). When treatment was performed in early and late periods of the multiplication cycle after OROV infection (time of addition assay), justicidin B and tuberculatin were shown to significantly reduce viral load, with inhibition greater than 99.99% (>4 log_10_) observed when treatment was initiated up to 6 h postinfection (hpi). Moreover, treatment of cells with the compounds at 12 hpi also resulted in a significant reduction (∼2 log_10_) when compared to the viral control, although less potent than the inhibition observed with treatment initiated up to 6 hpi. However, when treatment was initiated at 24 hpi, no statistical difference was observed between the viral load of treated and viral control (Figure 6d). This result demonstrated that the main mechanism of action of both arylnaphthalenes is in postentry of the virus into the host cell, in intracellular stages. In addition, the results of treatment with justicidin B or tuberculatin in the early and late periods did not show significant differences at any of the times evaluated.

**Figure 6:**
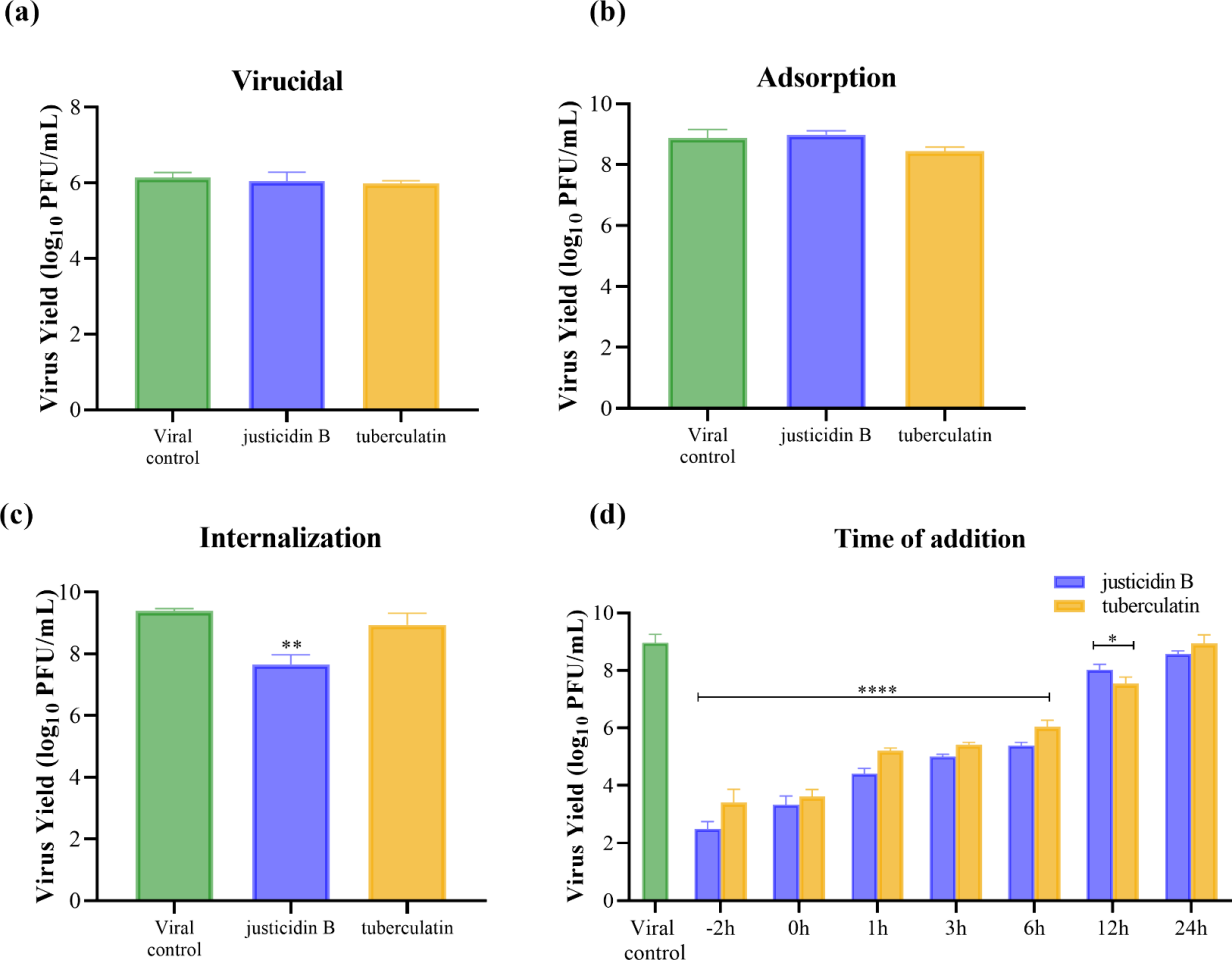
Justicidin B and tuberculatin inhibits OROV in postinfection stages. (a) Justicidin B and tuberculatin did not exhibit virucidal activity against OROV. (b) Treatment with justicidin B and tuberculatin during the viral adsorption stage did not result in a significant reduction in viral load. (c) Only justicidin B showed a significant reduction of 99% in viral load when added during the viral internalization stage. (d) Treatment with justicidin B and tuberculatin started up to 6 hpi showed a >99.99% reduction (>4 log_10_) in viral load, and the treatment after 12 hpi led to a ∼100-fold reduction (2 log_10_) in viral load. Data are presented as mean ± standard error of the mean (SEM) for all experiments, which “**” and “****” indicates a statistical difference (*P* < 0.01 and *P* < 0.0001) when compared to the viral control by using one-way ANOVA and Tukey’s post-test.

### 2.5 Molecular Docking Suggests Conserved Interactions of Justicidin B and Tuberculatin with OROV Gc Protein

Visual inspection of the justicidin B docking pose showed three predicted hydrogen interactions with LYS-601 (1.6 Å), MET-602 (2.1 Å), and TYR-620 (2.1 Å) (Figure 7A), suggesting a propensity of interaction between this compound with residues of the OROV Gc protein. Interestingly, the same interactions and distances were observed for tuberculatin (Figures 7B and 7C), suggesting that the same pose of interaction can contribute to the observed antiviral activity, which is consistent with the similar values obtained in our experimental assays (Table 1). As expected, chloroquine showed just one potential interaction with MET-602 (measured distance of 2.8 Å), suggesting a lower fitness within the protein binding site (Figure 7D).

**Figure 7:**
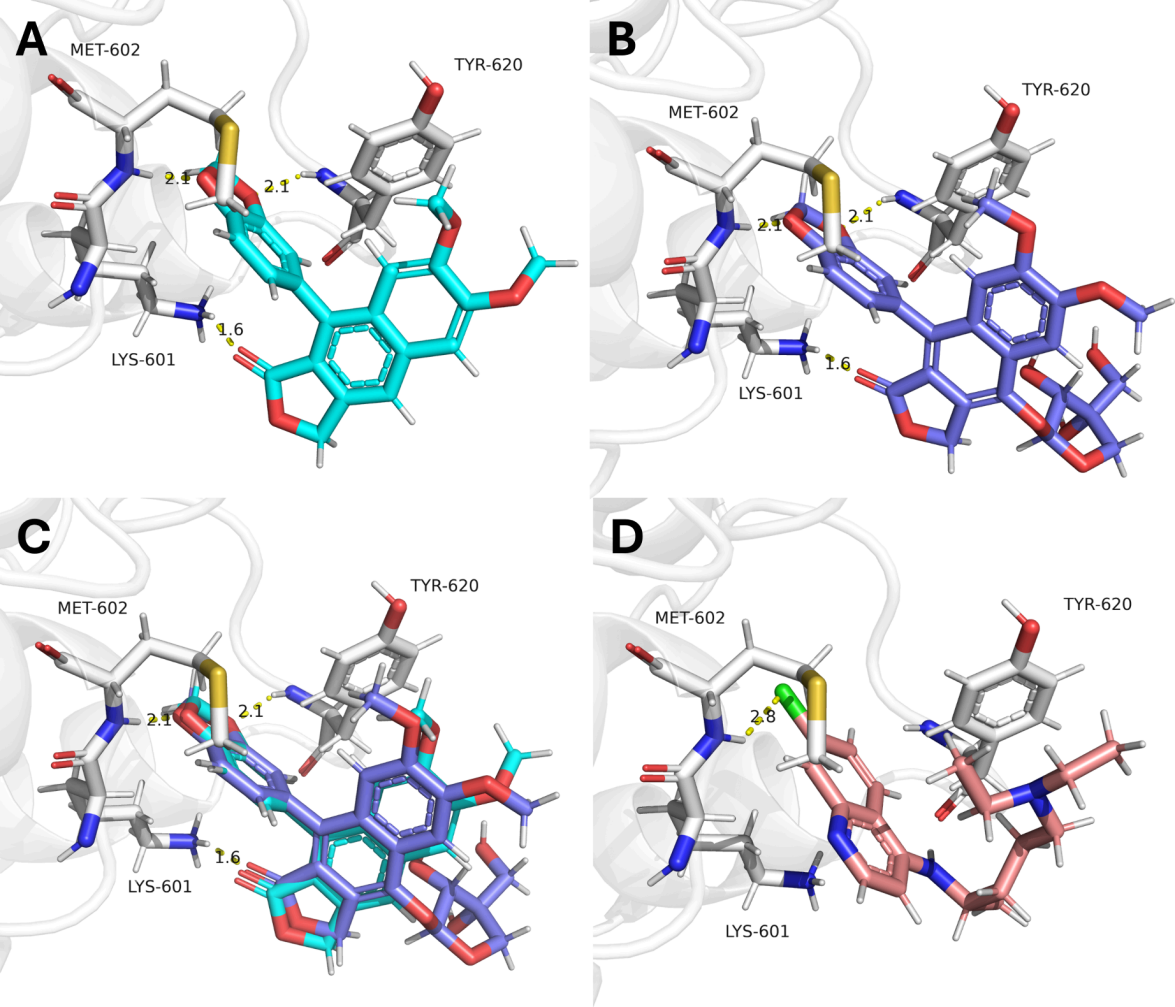
Justicidin B and tuberculatin are predicted to have higher fitness in OROV Gc protein than chloroquine. (A) Justicidin (cyan)-predicted pose to the OROV Gc (white) showed three predicted hydrogen interactions with LYS-601, MET-602, and TYR-620, equivalent to the (B) tuberculatin (slate) with the same predicted residues and a similar pose (C). As expected, (D) chloroquine (salmon)-predicted pose has a single potential interaction measured with MET-602. The protein structure is shown as a cartoon model with transparency, and residues are shown as sticks and labeled in black. All predicted interactions or measured distances (Å) are shown as yellow dashed lines. Images were generated using PyMOL (v2.5.7).

## 3 Discussion

The recent spread and increase in OROV outbreaks to new regions highlights the growing threat of Oropouche Fever amid climate change and a vulnerable population.^10^ OROV is still a poorly studied virus, which has attracted the attention of the scientific community in the last year, after unprecedented reports of cases of death, congenital infection resulting in miscarriage and/or anomalies involving the CNS with microcephaly, in addition to patients with relapse of symptoms after recovery.^11^ All these factors highlight the urgent need to improve surveillance, diagnostic capacity, and encourage research into the development of antivirals and vaccines that can reduce the impact of this emerging virus in an epidemic situation.

Plant-derived extracts are rich in bioactive compounds and are widely used in drug development, especially for the treatment of infectious diseases.^12^ In a previous study, *P. brasilensis* extracts were shown to be highly selective against OROV and other arboviruses.^9^ Thus, to contribute to research for the development of antivirals against OROV, we evaluated here two promising compounds isolated from *P. brasiliensis*, justicidin B and tuberculatin. Like the extracts, both lignans showed low toxicity in renal fibroblasts in vitro, confirming what we had previously described.^13^ Low cytotoxicity of justicidin B in normal PBMCs has also been demonstrated,^14^ while cytotoxicity studies in normal cells involving tuberculatin are limited. However, the cytotoxic effect (antiproliferative activity) of these arylnaphthalenes against different tumor cell lines is widely studied, especially when it comes to justicidin B.^15,16^

In antiviral assays, justicidin B and tuberculatin were shown to protect cells from OROV infection and reduce viral load effectively at low concentrations. The SI showed that the anti-OROV activity found for both compounds is highly selective, with justicidin B (SI = 2307) presenting an SI twice as high as that of tuberculatin (SI = 1153). SI is an important parameter to evaluate the therapeutic window of a drug candidate; the higher the SI, the greater the therapeutic potential, i.e, the safer and more selective the compound can be considered. High SI values (>100) drive the next stages of evaluation for a drug candidate.^34^ The selectivity found here for justicidin B and tuberculatin against OROV was considerably higher than that obtained in a previous study evaluating the extracts of leaves of *P. brasiliensis*, in which the SI ranged from 40 to 340.^9^ The authors also reported that the lignans annotated in the methanolic extract of *P. brasiliensis* leaves were identified as promising antiviral candidates by an in silico prediction, with tuberculatin detected in high relative concentration and having the highest activity score.^9^ Thus, our results corroborate previous in silico studies and show that justicidin B and tuberculatin are important active principles of *P. brasiliensis* extracts.

To date, few studies in the literature report compounds capable of inhibiting OROV. Recently, the anti-OROV activity of some broad-spectrum nucleoside analogs has been described. These include remdesivir, favipiravir, ribavirin evaluated using a recombinant OROV model expressing enhanced green fluorescent protein,^18^ and 4′-fluorouridine.^19^ Notably, both justicidin B and tubeculatin exhibited lower IC_50_ values than ribavirin, favipiravir, and remdesivir,^18^ suggesting greater antiviral potency compared to these referenced compounds. Furthermore, an in vivo study with interferon-α (IFN-α), which also has antiviral action against different viruses, showed that this cytokine has antiviral action against OROV only when used as a prophylactic treatment.^20^ Some other compounds showed anti-OROV activity in vitro, but with low selectivity, like quercetin hydrate (SI 9.3)^21^ and acridones (SI 70.83).^22^ These studies are examples of the urgency in prospecting compounds with anti-OROV activity with high selectivity and also draw attention to the potent and promising activity of justicidin B and tuberculatin. It is important to highlight that the infected cells and treated with justicidin B or tuberculatin at a concentration of 5 μg/mL presented cell viability close to that of the control without infection and without treatment, with no statistical difference, and were able to reduce the viral load by 99.99% (∼6 log_10_). In comparison with a recent study by Coimbra and collegues, lysergol demonstrated potent antiviral action against OROV in human hepatocyte cells (HuH-7.0), but the SI reached only 260 and the viral load reduction was approximately ∼ 5log_10_ (4.8 μM added 2 hpi).^23^ Furthermore, there are commercially available antivirals that reduce much less, such as remdesivir, a drug approved for treating Covid-19, that showed a ∼ 3 log_10_ reduction at 6.25 μM (3.8 μg/mL).^24^ Chloroquine (positive control) showed a viral load reduction of ∼5 log10 at a concentration of 32 μg/mL.^25^ These findings demonstrate once again that justicidin B and tuberculatin are far more potent and promising.

The greatest inhibition of viral load occurs at postentry stages for justicidin B and tuberculatin, particularly when treatment is initiated in the first hours postinfection (up to 6 hpi). Docking analysis further supports these findings, as both compounds showed a similar propensity to interact with residues of the OROV Gc glycoprotein. Given the essential role of Gc in mediating pH-dependent membrane fusion during viral entry,^26^ these interactions suggest that the compounds may interfere in early events, inhibiting Gc glycoprotein activity, preventing membrane fusion or genome release during cell infection. However, the pronounced antiviral effect observed at postentry stages indicates that additional steps of the viral cycle may also be affected, such as genome uncoating, translation, replication, virion assembly, and/or release. Additionally, the translation and replication of the *Peribunyaviridae* genome differ significantly from other viruses due to the segmented genome. In this sense, a compound with inhibitory activity at this stage would be a more specific compound for infections caused by *Peribunyaviridae*, as observed for hop compounds that appear to interact with the endonuclease domain of the L segment of OROV, which is essential for transcription, binding and cleavage of RNA.^27^ However, this does not appear to be the case for justicidin B, which has been identified as a broad-spectrum antiviral. Besides to OROV, low concentrations of justicidin B were also able to inhibit vesicular stomatitis virus (*Rhabdoviridae*),^28^ Sindibis (*Togaviridae*),^29^ and Zika (*Flaviviridae*).^13^ Viruses belong to four distinct families, with different replication mechanisms, but with one characteristic in common: they use receptor-mediated endocytosis (such as clathrin) to enter the host cell and require an acidic environment to activate the fusion of the endosomal membrane and viral envelope to release their genome into the cytoplasm. Besides this common point, studies have shown that justicidin B is capable of inhibiting clathrin-mediated endocytosis^30^ and that the analogue 7-hydroxyjusticidin B (diphylline) is a potent inhibitor of vacuolar (H^+^) ATPase, causing inhibition of endosomal acidification,^31^ which, consequently, interrupts the viral multiplication cycle. In addition to this evidence, the fact that justicidin B exhibits low antiviral activity against viruses that utilize both membrane fusion and endocytosis for entry, such as murine cytomegalovirus (*Herpesviridae*)^29^ and SARS-CoV-2 (*Coronaviridae*),^32^ supports our hypothesis regarding its antiviral mechanism of action.

We acknowledge that this study is limited to in vitro experiments, which represent only an initial step in the development of antiviral drugs. We emphasize that future studies are needed to comprehensively elucidate the mechanism of action of these lignans. In addition, we suggest further in vivo investigations, such as pharmacokinetic and toxicity studies, as well as translational approaches, such as human organ-on-a-chip models.

## 4 Conclusions

This study represents the second report of the in vitro antiviral activity of tuberculatin, a glycosylated analog of justicidin B, which has also demonstrated activity against the Zika virus.^13^ Furthermore, it is important to highlight that an efficient chemical synthesis of justicidin B and its derivatives has already been described,^33^ a fact that may contribute to a possible large-scale production. Finally, although further studies are needed, the set of results presented here strongly suggest that justicidin B and tuberculatin have promising therapeutic potential against Oropouche fever by reducing OROV’s ability to infect host cells. Moreover, our findings open perspectives for an antiviral evaluation in an animal model, in order to better characterize the pharmacological action of justicidin B and tuberculatin, which could contribute to the advancement of a therapeutic approach against Oropouche fever.

## 5 Methods

### 5.1 Isolation, Characterization, and Purity of Justicidin B and Tuberculatin

Justicidin B (compound 1) and tuberculatin (compound 2) were isolated from the methanolic extract of *P. brasiliensis* leaves, as previously described by Carvalho et al.^9^ and Ferraz et al.^13^ Briefly, dried and powdered leaves were extracted with methanol, and the crude extract was subjected to chromatographic fractionation using column chromatography followed by preparative HPLC. Structural identification of the isolated lignans was achieved by spectroscopic analyses, including ^1^H and ^13^C NMR and mass spectrometry (MS), with data compared to literature reports. In addition, untargeted LC–MS-based metabolomics analysis was previously performed to characterize the chemical profile of the extract, identifying lignans as major constituents, including justicidin B and tuberculatin.

The purity of the isolated compounds was determined by HPLC-DAD analysis and confirmed by NMR spectroscopy, showing chromatographic purities of 97% for justicidin B and 95% for tuberculatin. These purity levels are within the range commonly accepted for in vitro biological and antiviral assays.

### 5.2 Cell Lines and Viruses

Vero continuous lineage (ATCC CCL-81), derived from the African green monkey kidney (*Cercopithecus aethiops*), was used in all assays. Vero cells were cultured in Dulbecco’s Modified Eagle’s Minimal Medium (Sigma-Aldrich, USA) in a humidified incubator at 37 °C with 5% CO_2_. Media was supplemented with 5% fetal bovine serum (FBS; Sigma-Aldrich, USA), penicillin/streptomycin (200 U/mL), and amphotericin B (2.5 μg/mL) (Sigma-Aldrich, USA). Subculturing was performed when the cell monolayer reached 90 to 100% confluence. The OROV sample, Brazilian prototype strain BeAn 19991 [GenBank numbers KP052850 (segment L), KP052851 (segment M), and KP052852 (segment S)], was originally isolated from a vertebrate host (*Bradypus tridactylus*) in Pará State (Brazil) in 1960. The strain used in the assays was kindly provided by Professor Eurico de Arruda Neto, from the Institute of Virology Research of Universidade de São Paulo (USP). OROV stocks were prepared by infection of Vero cells at a multiplicity of infection (moi) of 0.01 and incubated at 37 °C for 2 days. The supernatant of infected cells was isolated and stored at −80 °C. Viral titration was carried out using the Dulbecco plate assay.

### 5.3 Cytotoxicity Assays

Cells were previously seeded in 96-well microplates (5 × 10^4^ cells/well). The protocol used was previously described.^13^ Briefly, cells were treated with justicidin B, tuberculatin, and chloroquine (positive control) at different concentrations and the plates were incubated at 37 °C for 48 h. The wells intended for cell control (CC), untreated cells, received only fresh culture medium. In addition, a vehicle control (DMSO) was also included. Cell viability was measured by the MTT (methylthiazolyldiphenyl-tetrazolium bromide, Sigma-Aldrich) colorimetric assay, and absorbance was measured in a spectrophotometer (VitorX3, PerkinElmer, Waltham, MA, USA) at 490 nm. The cytotoxic concentration for 50% of the cells (CC_50_) of each sample was determined using regression analysis, regarding the viability of untreated cells.

### 5.4 Antiviral Activity Assays

The antiviral evaluation of justicidin B and tuberculatin was carried out according to our previous study.^13^ For this, the 96-well microplates were previously seeded (5 × 10^4^ cells/well). A 2-fold dilution (ranging from 10 to 0.31 μg/mL) of justicidin B and tuberculatin was added to the cells, followed by infection with OROV at an moi of 1 (final volume of 200 μL/well). The cell control (CC) wells (cells untreated and uninfected) received only fresh culture medium, and the viral control (VC) wells were infected with OROV (cells infected and untreated). In addition, chloroquine was used as a positive control^25^ at concentrations ranging from 50 to 0.39 μg/mL and a negative control (DMSO - vehicle) was also included, followed by OROV infection (moi 1). The plate was incubated for 48 h at 37 °C, and aliquots of the supernatant from the treated wells, including the CC and VC, were made (stored at −80 °C until use). Cell viability was quantified by the MTT assay. The effective concentration for 50% of the cells (EC_50_) was calculated by regression analysis, expressed as the concentration that promoted the protection of 50% of the infected cells when compared to the viral control. The inhibitory concentration for 50% of the cells (IC_50_) was quantified by titration of the aliquots performed by the Dulbecco plate assay. The IC_50_ was expressed as the concentration of the antiviral compound required to reduce the OROV viral load by 50%. Furthermore, after 48 h of incubation, and before collecting the aliquots, the monolayers were photographed using an optical microscope at a magnification of 100× (Zeiss, Oberkochen, Germany). The selectivity index (SI) was calculated and expressed as the CC_50_/IC_50_ ratio.

### 5.5 Assessment of Antiviral Activity at Different Stages of Viral Infection

The antiviral mechanism of action of justicidin B and tuberculatin against OROV was evaluated using four main assays: virucidal, adsorption, internalization, and time of addition (early and late periods of the viral multiplication cycle). The methodology used was the same as that described previously by Ferraz et al.^13^ Briefly, virucidal activity was evaluated by incubating justicidin B or tuberculatin with OROV (5 × 10^6^ UFP) for 1 h at 37 °C, followed by 10–10,000-fold dilution to subinhibitory concentrations prior to infection. Viral quantification was determined by Dulbecco’s plaque formation assay. For adsorption assays, Vero cells (5 × 10^5^ cells/well, 12-well plates) were precooled (4 °C, 15 min) and infected with OROV in the presence of justicidin B or tuberculatin for 1 h at 4 °C. Cells were washed, fresh medium was added, and plates were incubated at 37 °C for 24 h. For internalization assays, cells were infected at 4 °C for 1 h, in the absence of the compounds, washed, and then treated with justicidin B or tuberculatin during a 1 h incubation at 37 °C. Noninternalized virions were inactivated using citrate buffer (pH 3). For time of addition assays, cells (5 × 10^4^ cells/well, 96-well plates) were infected and justicidin B or tuberculatin was added at – 2, 0, 1, 3, 6, 12, or 24 h postinfection. Viral titers were determined by Dulbecco’s plaque formation assay. For all assays, cells were treated with both compounds at 10 μg/mL and infected with OROV at moi 1.

### 5.6 Ligand and Structure Preparation, and Molecular Docking

Justicidin B, tuberculatin, and chloroquine were prepared as described.^17,35^ Briefly, the 3D structures were imported from PubChem and adjusted on Discovery Studio (BIOVIA, USA, 2017), where their stable conformational units,^36^ i.e., lowest energy, were manually generated using “clean geometry”. The OROV Glycoprotein Gc head domain (UniProt: A0A0D4BSW3), was selected from the Protein Data Bank^37^ (PDB ID: 6H3X;^38^ 2.09 Å resolution). The structure was prepared as described,^39^ using PDB 2PQR.^40^ Briefly, water molecules were removed, hydrogen atoms were added, missing side chains were fixed, and the force field (AMBER) was applied, while the protonation states of amino acids were optimized at pH 7.0 with PROPKA.^41^ Molecular docking was performed using GOLD^42^ v2025.1.0. To predict potential binding modes of the compounds (justicidin B, tuberculatin, and chloroquine). The *Schmallenberg Virus* Glycoprotein Gc head domain bond to the monoclonal antibody scFv 1C11 (PDB ID: 6H3T, 2.84 Å resolution) was used for comparison purposes considering the superimposed structures to select centered residues.^43^ Then, docking runs were performed as in a blind docking (i.e., considering a region based on the centered residues) as reported.^43^ The selected parameters were: (i) all protein rotatable bonds fixed; (ii) binding site defined from the selected centered residue (Arg597), within 20 Å; (iii) chemscore kinase as a template; (iv) 200 genetic algorithm runs; (v) CHEMPLP as the scoring function (no “early termination” and search option set as slow and “automatic”). The selected pose was obtained based on the highest scores (CHEMPLP fitness) and by visual inspection.^44^ Images were generated using the PyMOL software (v2.5.7).

### 5.7 Statistical Analyses

All assays were performed in triplicate, and the data were analyzed using GraphPad Prism version 8 and expressed as mean ± standard error of the mean (SEM). According to the Kolmogorov–Smirnov normality test, differences between groups were considered significant when *P*-value ≤ 0.05, using one-way ANOVA with Tukey’s post-test.

## Supplementary Material



## Notes

^1^H NMR spectrum for structural validation of justicidin B (Figure S1) and tuberculatin (Figure S2) and molecular docking (Figure S3 and Figure S4) (PDF)
