# Supplementary material for: Antiviral Mechanisms of Justicidin B and Tuberculatin from Phyllanthus brasiliensis against Oropouche Virus Infection
Source: ACS Omega. 2026 May 19;11(21):31101–10. doi: 10.1021/acsomega.6c00627 (PMC13234677; doi:10.1021/acsomega.6c00627)
Supplement: Supplementary file 1 [file ao6c00627_si_001.pdf]

ORIGINAL ARTICLE

Antiviral mechanisms of justicidin B and tuberculatin from *Phyllanthus brasiliensis* against Oropouche virus infection

Marília Bueno da Silva Menegatto[§a], Ariane Coelho Ferraz[§a], Rafaela Lameira Souza Lima[a], Allen René Ruiz Hernández[a], Pedro Henrique Guimarães[a], Pedro Alves Machado-Junior[a], Paulo Wender P. Gomes[b,c], Sônia das Graças Santa Rosa Pamplona[b], Edina Raquel Meneses Silva[d], Ellen Gonçalves de Oliveira[e] , Adriana Cotta Cardoso Reis[f], José Carlos de Magalhães[g], Geraldo Célio Brandão[f], Jordana Grazziela Alves Coelho-dos-Reis[e], Erna Geessien Kroon[e], Consuelo Yumiko Yoshioka e Silva[d], Milton Nascimento da Silva[b] and Cintia Lopes de Brito Magalhães[a,h*]

[a]Biological Sciences Post-Graduation Program, Center for Research in Biological Sciences, Federal University of Ouro Preto, Ouro Preto, Minas Gerais, Brazil
[b]Chemistry Post-Graduation Program, Institute of Exact and Natural Sciences, Federal University of Pará, Belém, Pará, Brazil
[c]Biodiversity and Biotechnology Post-Graduation Program (BIONORTE-Pará), Federal University of Pará, Belém, Pará, Brazil
[d]Pharmaceutical Sciences Post-Graduation Program, Institute of Health Sciences, Federal University of Pará, Belém, Pará, Brazil
[e]Microbiology Post-Graduation Program, Institute of Biological Sciences, Federal University of Minas Gerais, Belo Horizonte, Minas Gerais, Brazil
[f]Pharmaceutical Sciences Post-Graduation Program, School of Pharmacy, Federal University of Ouro Preto, Ouro Preto, Minas Gerais, Brazil
[g]Biotechnology Post-Graduation Program, Federal University of São João del-Rei, São João del-Rei, Minas Gerais, Brazil
[h]Biotechnology Post-Graduation Program, Center for Research in Biological Sciences, Federal University of Ouro Preto, Ouro Preto, Minas Gerais, Brazil

[§] The authors contributed equally and are joint first authors.

*Corresponding author: Cintia Lopes de Brito Magalhães, Universidade Federal de Ouro Preto, Departamento de Ciências Biológicas, Campus Universitário Morro do Cruzeiro, Ouro Preto, Minas Gerais, 35.400-000, Brazil. Tel: +55-31-3559-1259. E-mail: cintia.magalhaes@ufop.edu.br

Keywords: justicidin B, tuberculatin, Oropouche virus, antiviral.

Figure S1

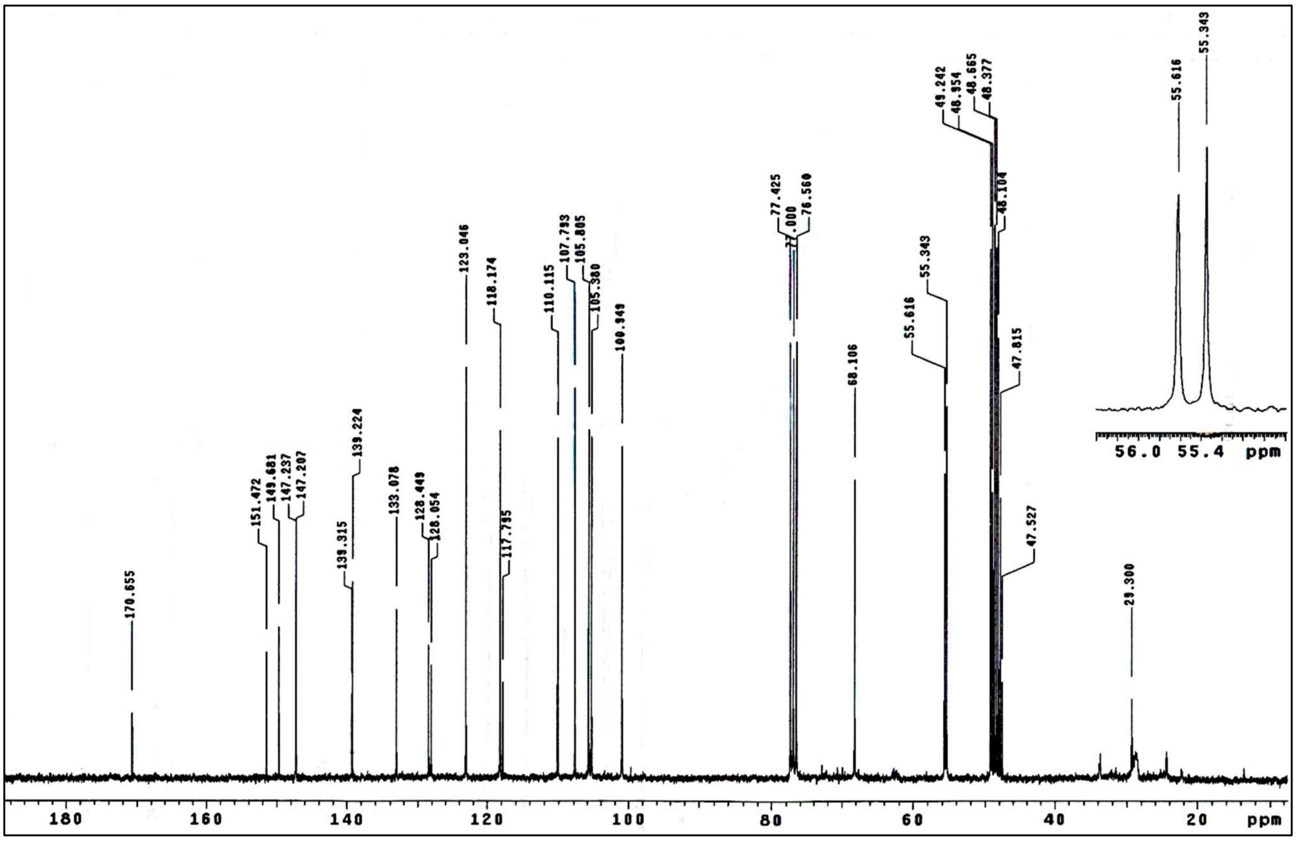

Figure S1. ^{13}**C NMR spectrum of justicidin B (75 MHz, $CDCl_3$ + CD_3OD).**

Figure S2

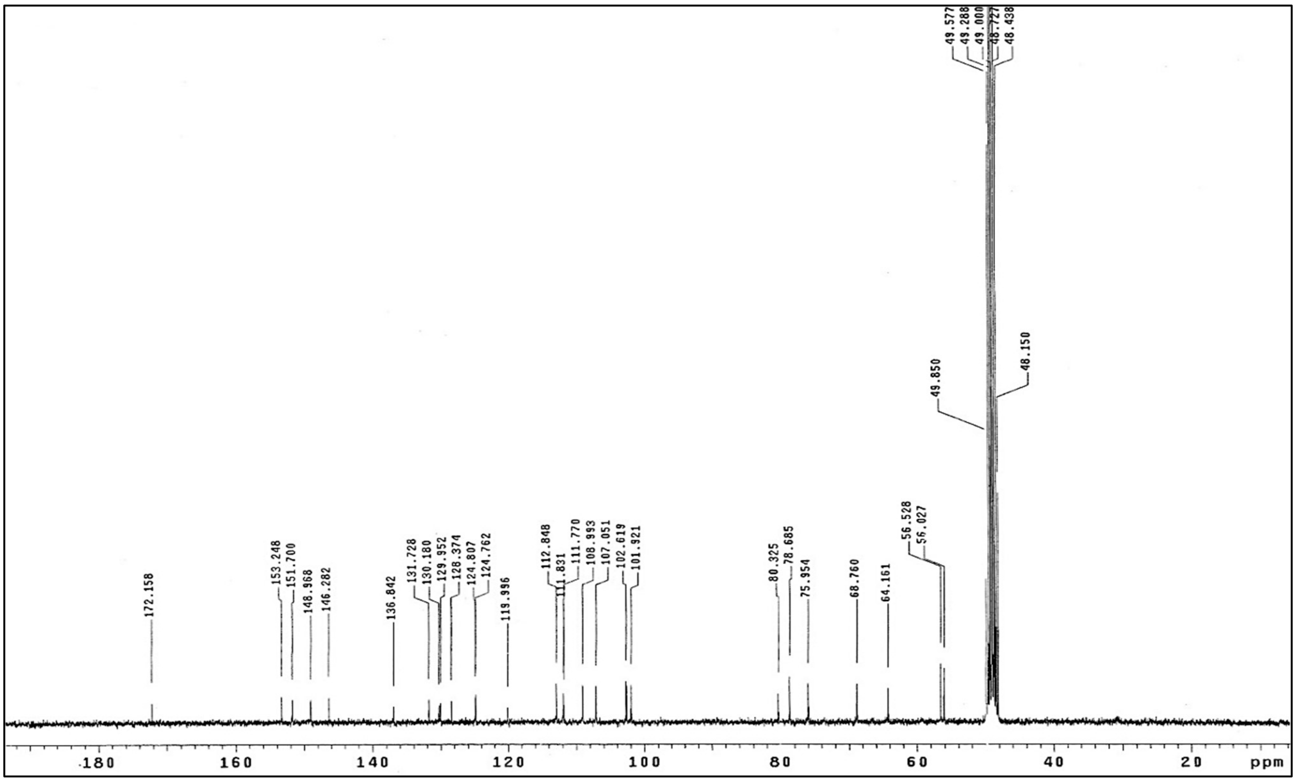

Figure S2. ^{13}C NMR spectrum of tuberculatin (75 MHz, CD_3OD).

Figure S3

Supplementary Table 2: Analysis of docking poses with RMSD < 2.0 Å, considering the cluster with the highest score pose:

**Compound**	**Cluster cutoff (< 2.0 Å)**	**N° Similar Pose Count**	**% of Total**
Justicidin B	1.84	57	28.5%
Tuberculatin	1.93	165	82.5%
Chloroquine	1.93	16	8%

*RMSD: Root-Mean-Square Deviation

To assess if the selected pose from visual inspection would account for the most frequent among the 200 docking solutions, an RMSD-based cluster analysis was performed using a 2.0 Å cutoff. The docking results for tuberculatin showed the highest convergence, with the most populated cluster containing 165 out of 200 solutions (82.5% of total poses). Justicidin B also exhibited a high convergence, with the largest cluster accounting for 28.5% of total poses (57 poses). Conversely, chloroquine showed the least convergence, with its major representative cluster containing only 16 poses (8% of the total).

Figure S4

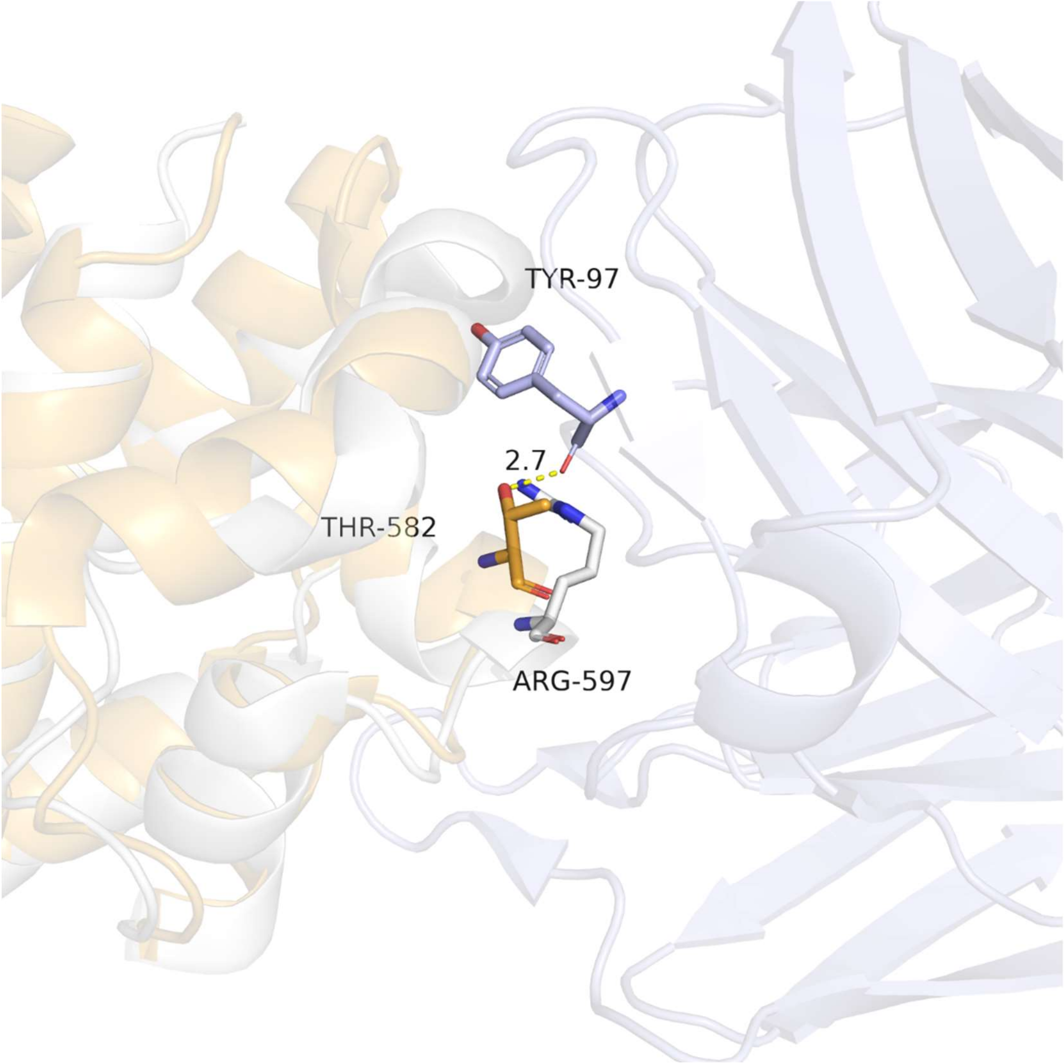

Figure S4. Molecular docking.
